# Supplementary material for: A BAC-guided haplotype assembly pipeline increases the resolution of the virus resistance locus CMD2 in cassava
Source: Genome Biol. 2025 Jun 29;26:185. doi: 10.1186/s13059-025-03620-8 (PMC12206362; doi:10.1186/s13059-025-03620-8)
Supplement: Supplementary file 1 — Additional file 1: Figs. S1–S2 and Tables S1–S6. [file 13059_2025_3620_MOESM1_ESM.docx]

# Supplemental Figures and Tables

**
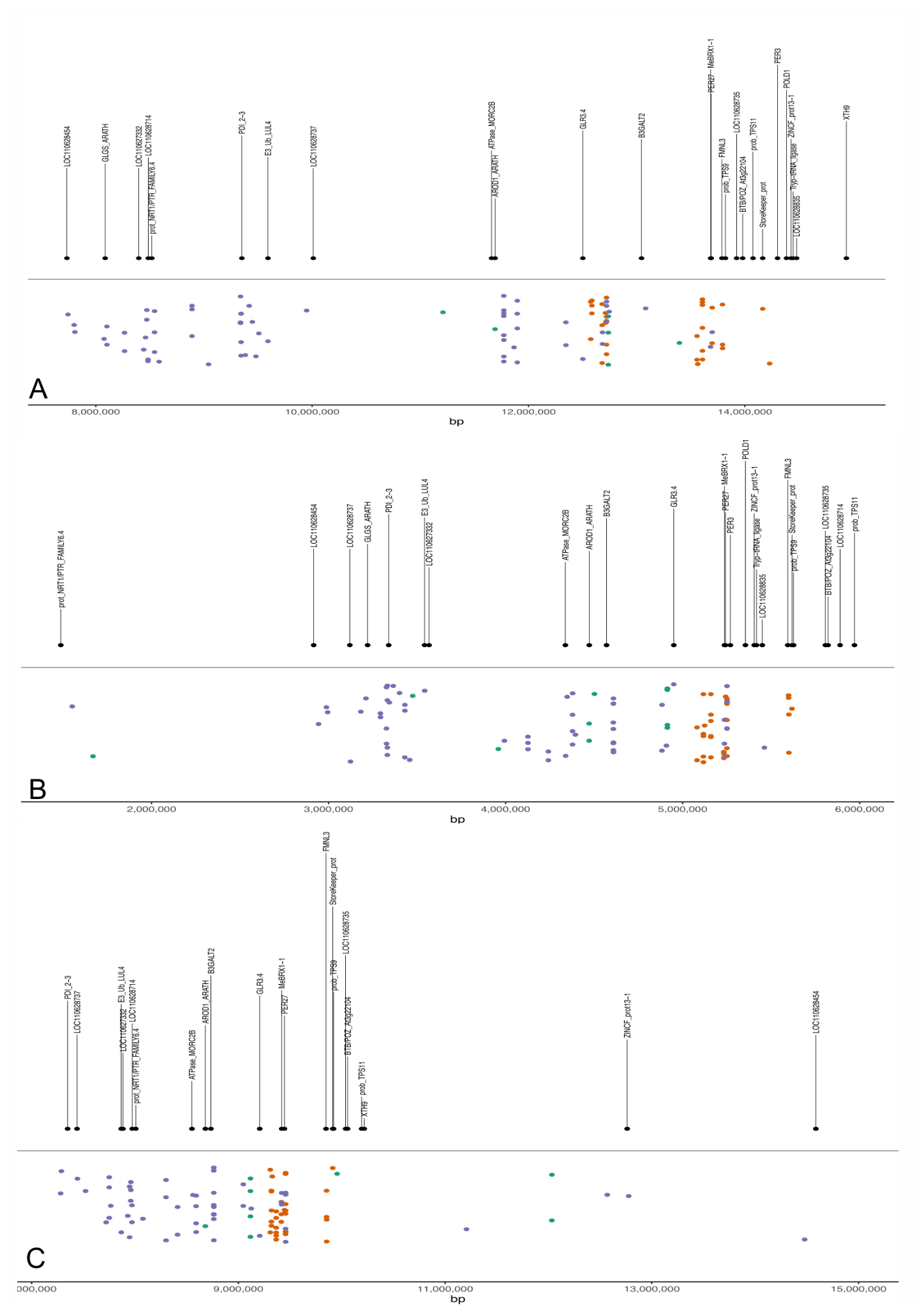
**

**Supplemental Figure 1: Markers and genes mapping on 60444 CMD2.** The *CMD2* locus in 60444 GCA_003957885.1 Kuon (A), 60444 GCA_963409065.1 Cornet haplotype 4632 (B), and 60444 GCA_963409065.1 Cornet haplotype 100051 (C). The black dots (above the horizontal black line) show the alignment positions of genes of interest that can be found in the *CMD2* region. The colored dots (below the horizontal black line) indicate various molecular markers associated with CMD resistance. The green dots indicate classical markers (RFLP and SSR markers) published by Akano et al. 2002 (43), Lokko et al. 2005 (44), Okogbenin et al. 2007 (45) and Okogbenin et al. 2012 (46). Orange dots indicate CMD2 SNP markers published by Rabbi et al. 2022 (12), and violet dots indicate markers published by Wolfe et al. 2016 (11). The x-axis of the plots indicate the base pair (bp) position on the genome.

**
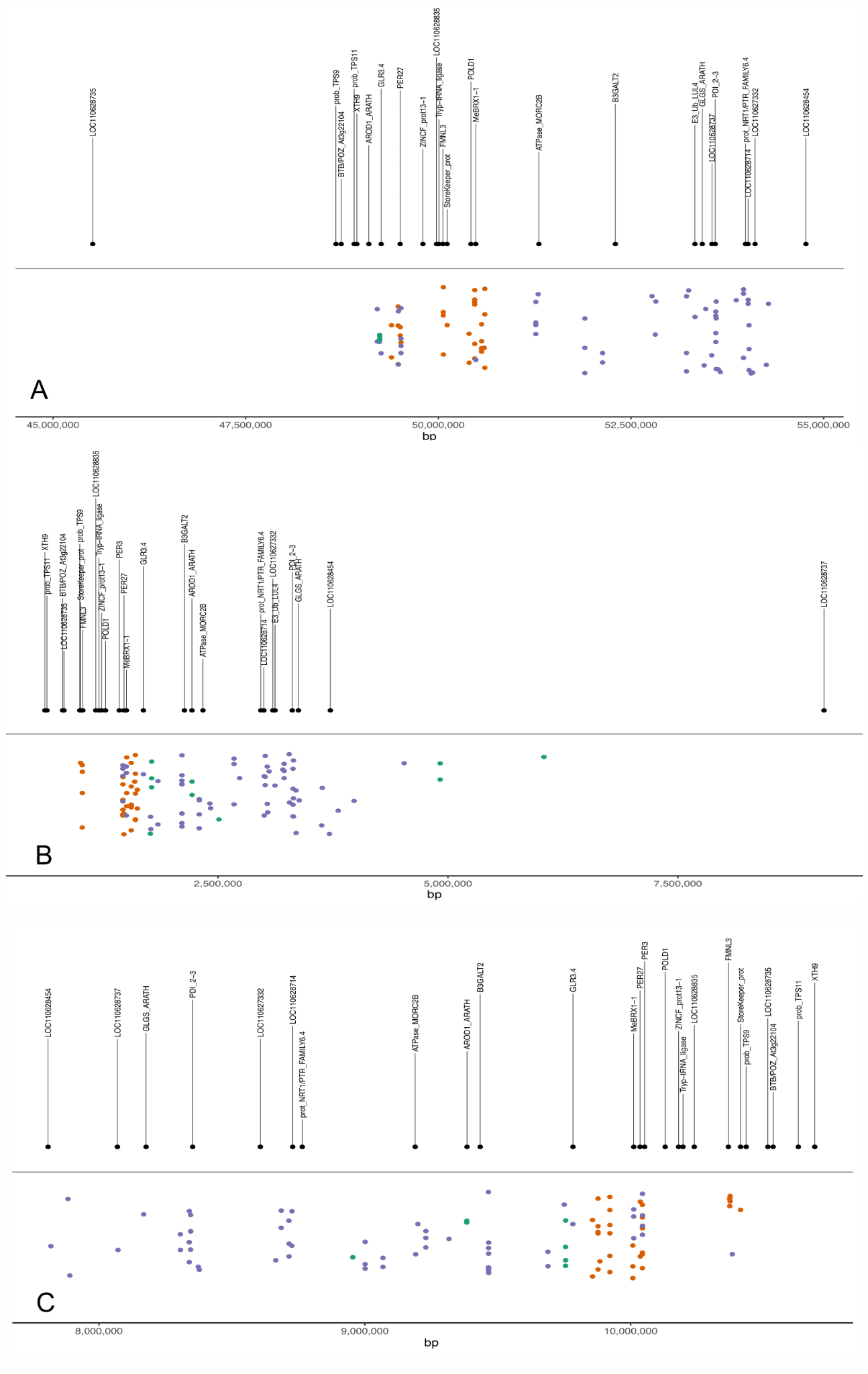
Supplemental Figure 2: Markers and genes mapping on *CMD2* from TME3 genome.** The CMD2 locus in TME3 GCA_003957885.1 Kuon (A), TME3 GCA_963409055.1 Cornet haplotype 100036 (B), and GCA_963409055.1 Cornet haplotype 100004 (C). The black dots (above the horizontal black line) show the alignment positions of genes of interest that can be found in the CMD2 region. The colored dots (below the horizontal black line) indicate various molecular markers associated with CMD resistance. The Green dots indicate classical markers (RFLP and SSR markers) published by Akano et al. 2002 (43), Lokko et al. 2005 (44), Okogbenin et al. 2007 (45) and Okogbenin et al. 2012 (46). Orange dots indicate CMD2 SNP markers published by Rabbi et al. 2022 (12), and violet dots indicate markers published by Wolfe et al. 2016 (11). The x-axis of the plots indicates the base pair (bp) position on the genome.

| **Probe** | **Primer** | **Sequence** |
| --- | --- | --- |
| PDI | PDI_F | ACAAGATGGACAAACGTCCT |
|  | PDI_R | TGAATGTGAAGAAAGGAGCA |
| Snp1 | snp1_F | TGAGCCATGAATGATGTTGTG |
|  | snp1_R | TGGTTGATGGAAGATCAGCA |
| Snp3 | snp3_F | AAATCTTGTTCGCGTCCTTC |
|  | snp3_R | GGTATCCAACTCCCCGAAAT |
| 169 | 169_F | ATCATCACCATGAGCAACGA |
|  | 169_R | CAATGGTCCTCAGGAATGCT |
| 158 | 158_F | GAGATCCACGATGATGCTGA |
|  | 158_R | TCAAAGCAACAAGCAAGGAA |
| PDI3600 | pdi3600_F | ATCACTAGCAGGGGCAAAAA |
|  | pdi3600_R | ACTTGCCAAATTTGATCTTCA |
| PDI3100 | pdi3100_F | TGTTACCAGGAAAAGAGTTCTGC |
|  | pdi3100_R | GCGAGCTGCAGTTGCTAAAT |
| 3 | 3_F | GTGGTTATTTGGCAACATGG |
|  | 3_R | TTGAATTTAGCATTCCGTTGAA |
| 32 | 32_F | GACAGCTGCTATACCTATCTTGG |
|  | 32_R | TAACTTTACCAATAAAAGCCAATCC |
| 80 | 80_F | TTATCTGGATATGGGGTGGA |
|  | 80_R | AAGGAAAAGGGGCAGGATTA |
| Z3 | Z3F | AAAGTTTGAGTTTCGGCTCTGTCC |
|  | Z3R | GAAAGACTATCTCACAACCGGG |
| Z7 | Z7F | TAGGGATTATGTCTTGGCTGTC |
|  | Z7R | CTTTAGTGTCAATTCCCTGTGG |
| Z9 | Z9F | CAACTCCTTTCCACTCCTCAATG |
|  | Z9R | CAAACCCAACAAGTCCAGCAGATT |
| Z12 | Z12F | CAGACAGCTTGTCCCTACTAAATC |
|  | Z12R | CCAGGAATTTCACTAGAAGAAGGG |
| Z16 | Z16F | CAACAGGAATCAAGTCACTCAC |
|  | Z16R | GGTTCTTCCTTCAACTCCTCTGAT |
| Z18 | Z18F | AGAGGTCTTCAGTTGGTTATGG |
|  | Z18R | CAAATCCAACCCAGGAACCTCTT |
| Z21 | Z21F | TTTGGGATACAGGGTTGCTTAG |
|  | Z21R | GATTTGTGAAGACGAAGGCTCT |
| Z22 | Z22F | AGCTCATGTGAGGGAGTTGGATAG |
|  | Z22R | CATCCTCCTCTCTGATTCTCAA |
| Z23 | Z23F | CACCTCCACCATACTATCCAAATC |
|  | Z23R | GCAGCAACTCCATAAACCTGTCA |
| Z30 | Z30F | TTTCCATCTTGACTCTGCCTCC |
|  | Z30R | CCATCTCAAGAAAGGTTGCTAC |
| Z36 | Z36F | ACCTTCAAGAACCAAGCTGATG |
|  | Z36R | GAGATGCAGGATATGTGCCTGA |
| Z37 | Z37F | AATAGACCTGTGCATCAGGGTG |
|  | Z37R | CTTGCTGCTTACCGAGTAGATGA |
| Z39 | Z39F | TGCATCTCAATCTCCTGGTATC |
|  | Z39R | GAATCGAGTCTCACCTACAAGAG |
| Z41 | Z41F | TAGCAGTTGCTGTGGGTTACAATG |
|  | Z41R | GAATGTCTGAAATGGGCTACTTGG |
| Z86 | Z86F | GGCTAATCCAGCAAGAAAGAGAACG |
|  | Z86R | CACCCGTAGAAAGAAAGACCCGAAT |
| Z87 | Z87F | CAACTGGAAGGAAGGATGACAGAG |
|  | Z87R | CAGCATTGATTTGTCCTTGTCC |
| Z91 | Z91F | TTATAGAGGTCAGGGTGGTTCTTGC |
|  | Z91R | CAGCCGCGTGAATCAACAGTAAAG |

**Supplemental Table 1: Primers used in this study for making BAC probes**

| **Genomes** | **BAC mapping percentage** | **BUSCO completeness %** |
| --- | --- | --- |
| **60444 with reads above 8kb, Flye default setting** | **81.45** | **72,9** |
| GCA_003957885.1 (60444) | 73.15 | 98,6 |
| 60444 all reads, Flye default setting | 81.40 | 70,2 |
| 60444 all reads,-L3000, wtdbg2 default setting | 53.09 | 77,5 |
| 60444 all reads,L10000, wtdbg2 default setting | 56.55 | 75,9 |
| 60444 all reads,L2000, wtdbg2 default setting | 59.81 | 11,3 |
| 60444 all reads,L5000, wtdbg2 default setting | 63.30 | 76,8 |
| 60444 all reads,-L8000, wtdbg2 default setting | 44.90 | 76,2 |
| 60444 all reads,L300, wtdbg2 default setting | 46.50 | 70,1 |
| 60444 all reads, CANU with stopOnLowCoverage=5 and cnsErrorRate=0.25 | 67.46 | 98 |
| 60444 with reads above 8kb, CANU with stopOnLowCoverage=5 and cnsErrorRate=0.25 | 62.34 | 88,3 |
| **TME3 with reads above 8kb, Flye default setting** | **80.02** | **95,8** |
| GCA_003957995.1 (TME3) | 61.08 | 98,2 |

**Supplemental Table 2: BAC mapping percentage and BUSCO completeness on genome assemblies.**

| **Genomes** | **BUSCO** | | **QUAST** | | | |
| --- | --- | --- | --- | --- | --- | --- |
|  | **Single** | **Duplication** | **Total length (Gb)** | **N50** | **#Contigs** | **N percentage** |
| cv. 60444 | 57 % | 15.9 % | 1,42 | 18240505 | 6402 | 16039.45 |
| TME 3 | 60.6 % | 35.2 % | 1,56 | 22737009 | 8161 | 16936.51 |
| GCA_003957885.1 (60444) | 61,2 % | 37 % | 1,27 | 59195861 | 4440 | 23496.76 |
| GCA_003957995.1 (TME3) | 55.2 % | 43.4 % | 1,22 | 53513187 | 5398 | 22496.91 |

**Supplemental Table 3: Statistics of genome assemblies.**

| **Gene_ID** | **prped_probability** | **60444_presence (GCA_963409065.1 Cornet)** | **TME3-NCBI_presence**  **(GCA_003957995.1 Kuon)** | **60444_Isoseq** | **TME3_Isoseq** | **CMD2_candidate** | **Length** | **Interproscan_domain** |
| --- | --- | --- | --- | --- | --- | --- | --- | --- |
| evm.model.Hap2-Super-Scaffold_100004.552 | 0.9010995973506256 | Out CMD2 contig | V | V | X | X | 868 | GluR_Plant  Periplasmic binding protein-like II  Receptor family ligand binding region  Bacterial extracellular solute-binding proteins- family 3  GLUTAMATE RECEPTOR 3.4  GluR_14  Ligand-gated ion channel  PBP1_GABAb_receptor_plant  IGluLR_plant  Periplasmic binding protein-like I  IONOTROPIC GLUTAMATE RECEPTOR |
| evm.model.Hap1-Super-Scaffold_100036.36 | 0.8557790799817562 | V | X | X | V | V | 896 | Ligand-gated ion channel  Periplasmic binding protein-like II  PBP1_GABAb_receptor_plant  Metabotropic gamma-aminobutyric acid type B receptor signature  IGluLR_plant  IONOTROPIC GLUTAMATE RECEPTOR  Receptor family ligand binding region  GLUTAMATE RECEPTOR 3.4  Periplasmic binding protein-like I  Bacterial extracellular solute-binding proteins- family 3  GluR_14  consensus disorder prediction |
| evm.model.Hap2-Super-Scaffold_100004.551 | 0.8395016558903129 | V | V | V | V | X | 911 | Bacterial extracellular solute-binding proteins- family 3  Receptor family ligand binding region  IGluLR_plant  GluR_Plant  IONOTROPIC GLUTAMATE RECEPTOR  GLUTAMATE RECEPTOR 3.7  PBP1_GABAb_receptor_plant  Prokaryotic membrane lipoprotein lipid attachment site profile.  Periplasmic binding protein-like I  Ligand-gated ion channel  Metabotropic gamma-aminobutyric acid type B receptor signature  Periplasmic binding protein-like II  Metabotropic glutamate GPCR signature |
| evm.model.Hap2-Super-Scaffold_100004.501 | 0.8114243528832168 | Out CMD2 contig | Out CMD2 contig | V | V | X | 821 | consensus disorder prediction  NODULIN HOMEOBOX |
| evm.model.Hap2-Super-Scaffold_100004.497 | 0.7893735297342348 | V | V | X | X | V | 589 | MEMBRANE PROTEIN |
| evm.model.Hap1-Super-Scaffold_100036.99 | 0.7733181488118763 | V | V | V | V | X | 925 | CBM20 (carbohydrate binding type-20) domain profile.  Ribonuclease E/G family  CBM_20_2  RIBONUCLEASE E/G-LIKE PROTEIN- CHLOROPLASTIC  RNaseEG: ribonuclease- Rne/Rng family  Nucleic acid-binding proteins  Immunoglobulins  Starch-binding domain-like |
| evm.model.Hap2-Super-Scaffold_100004.541 | 0.7270525556199235 | X | X | V | X | X | 729 | 35exoneu6  POLYMYOSITIS/SCLERODERMA AUTOANTIGEN-RELATED  Ribonuclease H-like  HRDC domain  HRDC-like  PROTEIN RRP6-LIKE 3  3'-5' exonuclease |
| evm.model.Hap2-Super-Scaffold_100004.545 | 0.7208088129583924 | V | V | X | X | V | 485 | Terpene synthase family- metal binding domain  Terpene Cyclase Like 1 C Terminal Domain  Farnesyl Diphosphate Synthase  Isoprenoid Synthase Type I  TERPENE SYNTHASE 12-RELATED  Terpene synthase- N-terminal domain  Terpenoid synthases  Terpenoid cyclases/Protein prenyltransferases  OS04G0344100 PROTEIN-RELATED |
| evm.model.Hap1-Super-Scaffold_100036.47 | 0.7152823677851056 | V | V | X | X | V | 408 | Terpene_cyclase_plant_C1  Terpene Cyclase Like 1 C Terminal Domain  OS04G0344100 PROTEIN-RELATED  Terpenoid cyclases/Protein prenyltransferases  Farnesyl Diphosphate Synthase  Isoprenoid Synthase Type I  Terpene synthase family- metal binding domain |
| evm.model.Hap2-Super-Scaffold_100004.525 | 0.7071564923891883 | X | X | V | V | X | 765 | Late exocytosis- associated with Golgi transport  Calcium-dependent channel- 7TM region- putative phosphate  consensus disorder prediction  Cytosolic domain of 10TM putative phosphate transporter  PROBABLE MEMBRANE PROTEIN DUF221-RELATED  consensus disorder prediction  PROTEIN OSCA1 |
| evm.model.Hap1-Super-Scaffold_100036.68 | 0.6951134205709454 | V | V | X | X | V | 754 | consensus disorder prediction  Late exocytosis- associated with Golgi transport  Calcium-dependent channel- 7TM region- putative phosphate  PROTEIN OSCA1  PROBABLE MEMBRANE PROTEIN DUF221-RELATED  Cytosolic domain of 10TM putative phosphate transporter |
| evm.model.Hap1-Super-Scaffold_100036.73 | 0.6541367694570865 | V | X | X | V | V | 562 | POT family  MFS general substrate transporter like domains  PROTEIN NRT1/ PTR FAMILY 6.4-LIKE  OLIGOPEPTIDE TRANSPORTER-RELATED |
| evm.model.Hap2-Super-Scaffold_100004.537 | 0.6116018128562075 | X | X | V | V | X | 366 | Prephenate dehydratase domain profile.  ACT_CM-PDT  Prephenate dehydratase signature 2.  PBP2_Ct-PDT_like  Periplasmic binding protein-like II  AROGENATE/PREPHENATE DEHYDRATASE  ACT domain profile.  ACT-like  Prephenate dehydratase  PREPHENATE DEHYDRATASE P PROTEIN |
| evm.model.Hap2-Super-Scaffold_100004.521 | 0.5979740774809379 | Out CMD2 contig | V | V | V | X | 589 | MFS general substrate transporter like domains  PROTEIN NRT1/ PTR FAMILY 6.4-LIKE  OLIGOPEPTIDE TRANSPORTER-RELATED  POT family  MFS general substrate transporter |
| evm.model.Hap1-Super-Scaffold_100036.62 | 0.5967214267801142 | Out CMD2 contig | X | V | V | X | 686 | CW-type Zinc Finger  Histidine kinase-- DNA gyrase B-- and HSP90-like ATPase  ZINC FINGER CW-TYPE COILED-COIL DOMAIN PROTEIN 3.  OS06G0622000 PROTEIN  consensus disorder prediction  ATPase domain of HSP90 chaperone/DNA topoisomerase II/histidine kinase  Morc6 ribosomal protein S5 domain 2-like |
| evm.model.Hap1-Super-Scaffold_100036.55 | 0.5716795773591182 | X | X | X | X | V | 879 | SNF2 family N-terminal domain  TRANSCRIPTION TERMINATION FACTOR 2-RELATED  RING/U-box  Zinc/RING finger domain  RING-type zinc-finger  Superfamilies 1 and 2 helicase ATP-binding type-1 domain profile.  Superfamilies 1 and 2 helicase C-terminal domain profile.  SF2_C_SNF  consensus disorder prediction  DEXDc_SHPRH-like  helicmild6  P-loop containing nucleoside triphosphate hydrolases  ultradead3  Helicase conserved C-terminal domain  ring_2 |
| evm.model.Hap2-Super-Scaffold_100004.507 | 0.5694020177734762 | V | V | V | V | X | 437 | cax: calcium/proton exchanger  VACUOLAR CALCIUM ION TRANSPORTER  Sodium/calcium exchanger protein  caca2: calcium/proton exchanger |
| evm.model.Hap2-Super-Scaffold_100004.539 | 0.5426593147869594 | V | V | X | X | V | 351 | PROSTAGLANDIN REDUCTASE  N-terminal domain of oxidoreductase  NAD(P)-binding Rossmann-fold domains  2-ALKENAL REDUCTASE (NADP(+)-DEPENDENT)-LIKE  Zinc-binding dehydrogenase  GroES-like  PKS_ER_names_mod |
| evm.model.Hap2-Super-Scaffold_100004.542 | 0.5280101953343767 | X | X | X | X | V | 529 | Farnesyl Diphosphate Synthase  Isoprenoid Synthase Type I  TERPENE SYNTHASE 12-RELATED  Terpene synthase family- metal binding domain  Terpene_cyclase_plant_C1  Terpenoid synthases  Farnesyl Diphosphate Synthase  Terpene synthase- N-terminal domain  OS04G0344100 PROTEIN-RELATED  Terpene Cyclase Like 1 C Terminal Domain  Terpenoid cyclases/Protein prenyltransferases |
| evm.model.Hap2-Super-Scaffold_100004.532 | 0.5277047250642454 | V | V | X | V | V | 613 | Morc6 ribosomal protein S5 domain 2-like  ZINC FINGER CW-TYPE COILED-COIL DOMAIN PROTEIN 3.  Histidine kinase-- DNA gyrase B-- and HSP90-like ATPase  ATPase domain of HSP90 chaperone/DNA topoisomerase II/histidine kinase  OS06G0622000 PROTEIN |
| evm.model.Hap1-Super-Scaffold_100036.43 | 0.5 | X | X | V | V | X | 951 | LeuD/IlvD-like  ACONITASE/IRON-RESPONSIVE ELEMENT FAMILY MEMBER  aconitase_1: aconitate hydratase 1  Aconitase family signature 1.  Aconitase C-terminal domain  Aconitase family (aconitate hydratase)  Aconitase iron-sulfur domain  Aconitase family signature 2. |
| evm.model.Hap1-Super-Scaffold_100036.94.1.61231383 | 0.492551118337242 | Out CMD2 contig | Out CMD2 contig | V | V | X | 414 | Sodium/calcium exchanger protein  caca2: calcium/proton exchanger  cax: calcium/proton exchanger  VACUOLAR CATION/PROTON EXCHANGER  VACUOLAR CALCIUM ION TRANSPORTER |
| evm.model.Hap1-Super-Scaffold_100036.75 | 0.4908418454937844 | Out CMD2 contig | V | V | V | X | 539 | BNAC03G34680D PROTEIN  NAD(P)-binding Rossmann-fold domains  Aminoacid dehydrogenase-like- N-terminal domain  aroE: shikimate dehydrogenase  Shikimate dehydrogenase substrate binding domain  Shikimate 5'-dehydrogenase C-terminal domain  Leucine Dehydrogenase  SHIKIMATE DEHYDROGENASE  Shikimate / quinate 5-dehydrogenase  DHQase_I  Aldolase class IAldolase  aroD: 3-dehydroquinate dehydratase- type I  Type I 3-dehydroquinase  NAD_bind_Shikimate_DH |
| evm.model.Hap1-Super-Scaffold_100036.87 | 0.47638444196707236 | V | V | V | V | X | 677 | E3 UBIQUITIN-PROTEIN LIGASE COP1  WD40 repeat-like  Zinc finger RING-type profile.  ring_2  RING-HC_COP1  Zinc/RING finger domain  Trp-Asp (WD) repeats signature.  RING/U-box  Zinc finger RING-type signature.  WD40  Trp-Asp (WD) repeats profile.  consensus disorder prediction  Coil  Zinc finger- C3HC4 type (RING finger)  WD domain- G-beta repeat |
| evm.model.Hap1-Super-Scaffold_100036.51 | 0.4723144809183478 | V | X | X | X | V | 312 | 2-ALKENAL REDUCTASE (NADP(+)-DEPENDENT)-LIKE  GroES-like  NAD(P)-binding Rossmann-fold domains  PROSTAGLANDIN REDUCTASE  PKS_ER_names_mod  Zinc-binding dehydrogenase |
| evm.model.Hap1-Super-Scaffold_100036.57 | 0.4717107073085255 | X | X | X | X | V | 607 | ZINC FINGER CW-TYPE COILED-COIL DOMAIN PROTEIN 3.  Zinc finger CW-type profile.  ATPase domain of HSP90 chaperone/DNA topoisomerase II/histidine kinase  OS06G0622000 PROTEIN  consensus disorder prediction |
| evm.model.Hap1-Super-Scaffold_100036.79 | 0.4441556977397326 | V | V | V | V | X | 611 | G1/S-SPECIFIC CYCLIN-E PROTEIN  consensus disorder prediction  OS05G0597400 PROTEIN |
| evm.model.Hap1-Super-Scaffold_100036.45 | 0.43873609978498446 | X | X | X | X | V | 505 | TERPENE SYNTHASE 12-RELATED  Terpene_cyclase_plant_C1  Terpene synthase- N-terminal domain  Terpene synthase family- metal binding domain  Farnesyl Diphosphate Synthase  Terpenoid synthases  OS04G0344100 PROTEIN-RELATED  TERPENE SYNTHASE 12-RELATED  Terpenoid cyclases/Protein prenyltransferases |
| evm.model.Hap2-Super-Scaffold_100004.514 | 0.4285325850314853 | X | X | X | X | V | 583 | G1/S-SPECIFIC CYCLIN-E PROTEIN  OS05G0597400 PROTEIN |
| evm.model.Hap2-Super-Scaffold_100004.534 | 0.4245975125511343 | V | V | X | X | V | 759 | P-loop containing nucleoside triphosphate hydrolases  TRANSCRIPTION TERMINATION FACTOR 2-RELATED  DEXDc_SHPRH-like  ultradead3  P-loop containing nucleoside triphosphate hydrolases  consensus disorder prediction  Superfamilies 1 and 2 helicase ATP-binding type-1 domain profile. SNF2 family N-terminal domain |
| evm.model.Hap1-Super-Scaffold_100036.81 | 0.41764803912629644 | X | X | X | X | V | 372 | ARM REPEAT SUPERFAMILY PROTEIN  consensus disorder prediction |
| evm.model.Hap1-Super-Scaffold_100036.44 | 0.4174312036070787 | V | X | X | X | V | 394 | Terpene synthase family- metal binding domain  TERPENE SYNTHASE 12-RELATED  Terpenoid cyclases/Protein prenyltransferases  Farnesyl Diphosphate Synthase  OS04G0344100 PROTEIN-RELATED  Terpene synthase- N-terminal domain  Terpenoid synthases |
| evm.model.Hap2-Super-Scaffold_100004.494 | 0.4129948869252456 | V | V | X | V | V | 876 | consensus disorder prediction  OS06G0608100 PROTEIN |
| evm.model.Hap1-Super-Scaffold_100036.49.1.612312ec | 0.40287570704406056 | V | V | V | V | X | 862 | Ribonuclease H-like  35exoneu6  3'-5' exonuclease  HRDC domain  consensus disorder prediction  PROTEIN RRP6-LIKE 3  POLYMYOSITIS/SCLERODERMA AUTOANTIGEN-RELATED |
| evm.model.Hap2-Super-Scaffold_100004.502 | 0.39753655150895717 | Out CMD2 contig | Out CMD2 contig | X | X | V | 568 | E3 UBIQUITIN-PROTEIN LIGASE KEG-LIKE  Transferase(Phosphotransferase) domain 1  consensus disorder prediction  Protein kinase domain profile.  Protein kinase-like (PK-like)  OS06G0639500 PROTEIN |
| evm.model.Hap2-Super-Scaffold_100004.505 | 0.3967982847427726 | Out CMD2 contig | Out CMD2 contig | V | V | X | 367 | BNAA08G30680D PROTEIN  Triose-phosphate Transporter family  SOLUTE CARRIER FAMILY 35 |
| evm.model.Hap2-Super-Scaffold_100004.512 | 0.36334890110022694 | V | V | V | V | X | 580 | ARM repeat  consensus disorder prediction  ARM REPEAT SUPERFAMILY PROTEIN |
| evm.model.Hap1-Super-Scaffold_100036.52.1.61231304 | 0.33391892158072123 | V | V | X | V | V | 252 | Prephenate dehydratase signature 2.  Periplasmic binding protein-like II  ACT_CM-PDT  ACT domain profile.  PREPHENATE DEHYDRATASE P PROTEIN  Prephenate dehydratase domain profile.  AROGENATE/PREPHENATE DEHYDRATASE  PBP2_Ct-PDT_like |
| evm.model.Hap2-Super-Scaffold_100004.555 | 0.30197426014393786 | Out CMD2 contig | Out CMD2 contig | V | V | X | 710 | HOX_1  Bet v1-like  'Homeobox' domain signature.  START_ArGLABRA2_like  Bet v1-like  START domain profile.  HOMEOBOX-LEUCINE ZIPPER PROTEIN MERISTEM L1  consensus disorder prediction  START_1  Coil |
| evm.model.Hap1-Super-Scaffold_100036.84 | 0.2985079020042521 | V | V | V | V | X | 194 | OXOGLUTARATE/IRON-DEPENDENT DIOXYGENASE  1-AMINOCYCLOPROPANE-1-CARBOXYLATE OXIDASE  Clavaminate synthase-like Coil |
| evm.model.Hap2-Super-Scaffold_100004.562 | 0.27981425800892473 | V | V | X | X | V | 329 | secretory_peroxidase  Plant peroxidase signature  Haem peroxidase superfamily signature  Peroxidases proximal heme-ligand signature.  Plant heme peroxidase family profile.  PEROXIDASE 25-RELATED  Peroxidases active site signature.  Heme-dependent peroxidases |
| evm.model.Hap1-Super-Scaffold_100036.100 | 0.2759093145551588 | V | V | V | V | X | 529 | OS07G0633600 PROTEIN |
| evm.model.Hap1-Super-Scaffold_100036.70 | 0.2625726676456461 | X | X | X | X | V | 270 | Protein tyrosine and serine/threonine kinase  Phosphorylase Kinase- domain 1  CHITIN ELICITOR RECEPTOR KINASE 1-RELATED  Transferase(Phosphotransferase) domain 1  Protein kinase-like (PK-like)  ILK  Serine/Threonine protein kinases active-site signature.  Protein kinase domain profile.  serkin_6 |
| evm.model.Hap2-Super-Scaffold_100004.540 | 0.2578879414476308 | Out CMD2 contig | Out CMD2 contig | V | V | X | 395 | Galactosyltransferase  BETA-1-3-N-ACETYLGLUCOSAMINYLTRANSFERASE  Domain of unknown function (DUF4094)  HEXOSYLTRANSFERASE  Coil |
| evm.model.Hap1-Super-Scaffold_100036.59 | 0.24867590395999326 | X | X | X | X | V | 208 | Protein kinase domain profile.  MAP KINASE KINASE KINASE  Transferase(Phosphotransferase) domain 1  CCR4-NOT TRANSCRIPTIONAL COMPLEX SUBUNIT CAF120-RELATED  serkin_6  Protein kinase-like (PK-like)  Transferase(Phosphotransferase) domain 1 |
| evm.model.Hap2-Super-Scaffold_100004.490 | 0.24129512153502283 | V | V | V | V | X | 542 | OS07G0633600 PROTEIN |
| evm.model.Hap2-Super-Scaffold_100004.530 | 0.23547113417844592 | X | X | X | X | V | 392 | Protein kinase domain  Protein kinases ATP-binding region signature.  Transferase(Phosphotransferase) domain 1  Protein kinase domain profile.  MITOGEN-ACTIVATED PROTEIN KINASE KINASE KINASE 15  Protein kinase-like (PK-like)  serkin_6  STKc_MAPKKK  CCR4-NOT TRANSCRIPTIONAL COMPLEX SUBUNIT CAF120-RELATED |
| evm.model.Hap2-Super-Scaffold_100004.524 | 0.21552478682001472 | X | X | X | X | V | 577 | Transferase(Phosphotransferase) domain 1  CHITIN ELICITOR RECEPTOR KINASE 1  LysM domain profile.  Protein kinases ATP-binding region signature.  Protein tyrosine and serine/threonine kinase  serkin_6  TonB-dependent receptor proteins signature 1.  LysM  Phosphorylase Kinase- domain 1  CHITIN ELICITOR RECEPTOR KINASE 1-RELATED  Serine/Threonine protein kinases active-site signature.  Protein kinase-like (PK-like)  LysM_2  Protein kinase domain profile. |
| evm.model.Hap1-Super-Scaffold_100036.104.1.612313d1 | 0.2112464684253818 | V | V | V | V | X | 284 | Coil  IGPS  Ribulose-phoshate binding barrel  TRYPTOPHAN BIOSYNTHESIS PROTEIN  ALDOLASE-TYPE TIM BARREL FAMILY PROTEIN-RELATED  Aldolase class I  Indole-3-glycerol phosphate synthase signature. |
| evm.model.Hap1-Super-Scaffold_100036.64 | 0.20910290552304106 | V | V | X | X | V | 427 | Phosphorylase Kinase- domain 1  Protein kinases ATP-binding region signature.  serkin_6  Protein kinase-like (PK-like)  CCR4-NOT TRANSCRIPTIONAL COMPLEX SUBUNIT CAF120-RELATED  Transferase(Phosphotransferase) domain 1  Protein kinase domain  MITOGEN-ACTIVATED PROTEIN KINASE KINASE KINASE 15  STKc_MAPKKK |
| evm.model.Hap1-Super-Scaffold_100036.46 | 0.19561513682528847 | V | V | V | V | X | 276 | Activating enzymes of the ubiquitin-like proteins  ThiF family  UBIQUITIN-ACTIVATING ENZYME E1  NEDD8-ACTIVATING ENZYME E1 REGULATORY SUBUNIT |
| evm.model.Hap1-Super-Scaffold_100036.33 | 0.18864454807486822 | V | V | V | V | X | 647 | HOX_1  Bet v1-like  Homeodomain-like  START_ArGLABRA2_like  Bet v1-like  'Homeobox' domain profile.  START domain profile.  consensus disorder prediction  HOMEOBOX-LEUCINE ZIPPER PROTEIN MERISTEM L1  START domain  Coil |
| evm.model.Hap1-Super-Scaffold_100036.37 | 0.18542350860571996 | X | X | X | X | V | 632 | Ligand-gated ion channel  Receptor family ligand binding region  IONOTROPIC GLUTAMATE RECEPTOR  Voltage-gated potassium channels  GluR_14  GLUTAMATE RECEPTOR 3.7  Periplasmic binding protein-like II |
| evm.model.Hap2-Super-Scaffold_100004.519 | 0.17898179872411138 | V | X | V | X | X | 500 | Type I 3-dehydroquinase  Aldolase  Shikimate dehydrogenase substrate binding domain  Aminoacid dehydrogenase-like- N-terminal domain  NAD_bind_Shikimate_DH  NAD(P)-binding Rossmann-fold domains  DHQase_I  Shikimate 5'-dehydrogenase C-terminal domain  Aldolase class I  BNAC03G34680D PROTEIN  Leucine Dehydrogenase |
| evm.model.Hap1-Super-Scaffold_100036.50 | 0.1768013327966887 | V | V | X | V | V | 403 | BETA-1-3-N-ACETYLGLUCOSAMINYLTRANSFERASE  Coil  HEXOSYLTRANSFERASE  Galactosyltransferase |
| evm.model.Hap2-Super-Scaffold_100004.517 | 0.172929061679052 | V | V | X | X | V | 234 | ACYL-MALONYL CONDENSING ENZYME-RELATED  SOLUTE CARRIER FAMILY 35 MEMBER G1  Multidrug resistance efflux transporter EmrE |
| evm.model.Hap1-Super-Scaffold_100036.72 | 0.17067961363051942 | V | X | V | V | X | 177 | Zinc/RING finger domain  OS08G0421900 PROTEIN  FYVE/PHD zinc finger |
| evm.model.Hap1-Super-Scaffold_100036.101 | 0.1693169882843195 | V | V | V | V | X | 367 | Armadillo/plakoglobin ARM repeat profile.  ARM REPEAT SUPERFAMILY PROTEIN  U BOX DOMAIN-CONTAINING  Kinesin-associated protein (KAP)  arm_5 |
| evm.model.Hap2-Super-Scaffold_100004.520 | 0.16723068248083853 | X | X | X | X | V | 504 | ENDO-1-4-BETA-GLUCANASE  ENDOGLUCANASE 11  Glycosyl hydrolase family 9  Six-hairpin glycosidases |
| evm.model.Hap1-Super-Scaffold_100036.77 | 0.1633679039256505 | V | V | X | X | V | 520 | Permease family  XANTHINE-URACIL / VITAMIN C PERMEASE FAMILY MEMBER  NUCLEOBASE-ASCORBATE TRANSPORTER 2 |
| evm.model.Hap1-Super-Scaffold_100036.61 | 0.14990874525279485 | V | V | V | X | X | 222 | Coil  BCR-ASSOCIATED PROTEIN- BAP  B-CELL RECEPTOR-ASSOCIATED 31-LIKE PROTEIN-RELATED |
| evm.model.Hap1-Super-Scaffold_100036.92 | 0.14846021552347244 | V | X | V | V | X | 376 | Glutaredoxin  Thioredoxin-like  PROTEIN DISULFIDE-ISOMERASE 2-3  pdi_dom: protein disulfide-isomerase domain  Thioredoxin family active site.  PDI_a_P5  P5_C |
| evm.model.Hap1-Super-Scaffold_100036.76 | 0.14564608057056844 | V | V | X | V | V | 398 | EamA-like transporter family  ACYL-MALONYL CONDENSING ENZYME-RELATED  Multidrug resistance efflux transporter EmrE  SOLUTE CARRIER FAMILY 35 MEMBER G1 |
| evm.model.Hap2-Super-Scaffold_100004.558 | 0.14079457843964588 | V | V | X | X | V | 186 | LATE EMBRYOGENESIS ABUNDANT (LEA) HYDROXYPROLINE-RICH GLYCOPROTEIN FAMILY |
| evm.model.Hap2-Super-Scaffold_100004.523 | 0.13604313969600076 | Out CMD2 contig | V | V | V | X | 189 | FYVE/PHD zinc finger  OS08G0421900 PROTEIN  BAH domain  Zinc/RING finger domain  CHROMATIN REMODELING PROTEIN EBS-LIKE  BAH domain profile.  BAH_4 |
| evm.model.Hap1-Super-Scaffold_100036.67 | 0.1348187373584988 | X | X | V | V | X | 484 | Cytochrome P450  CYTOCHROME P450 26  INACTIVE LINOLENATE HYDROPEROXIDE LYASE-RELATED  consensus disorder prediction  Cytochrome P450  E-class P450 group IV signature |
| evm.model.Hap2-Super-Scaffold_100004.485 | 0.12939014313506328 | X | X | V | V | X | 351 | Coil  ALDOLASE-TYPE TIM BARREL FAMILY PROTEIN-RELATED  Aldolase class I  TRYPTOPHAN BIOSYNTHESIS PROTEIN  Indole-3-glycerol phosphate synthase  Ribulose-phoshate binding barrel  TRYPTOPHAN BIOSYNTHESIS PROTEIN  IGPS  Indole-3-glycerol phosphate synthase signature. |
| evm.model.Hap1-Super-Scaffold_100036.48 | 0.1255629338676746 | V | V | V | V | X | 173 | UBIQUITIN-ACTIVATING ENZYME E1  Activating enzymes of the ubiquitin-like proteins  NEDD8-ACTIVATING ENZYME E1 REGULATORY SUBUNIT |
| evm.model.Hap2-Super-Scaffold_100004.500 | 0.12330663871400556 | V | X | X | V | V | 523 | ADP-glucose pyrophosphorylase signature 3.  LbH_G1P_AT_C  GLUCOSE-1-PHOSPHATE ADENYLYLTRANSFERASE  Nucleotide-diphospho-sugar transferases  ADP-glucose pyrophosphorylase signature 2.  Spore Coat Polysaccharide Biosynthesis Protein SpsA- Chain A  glgC: glucose-1-phosphate adenylyltransferase  Hexapeptide repeat proteins  Trimeric LpxA-like enzymes  ADP_Glucose_PP  Nucleotidyl transferase  ADP-glucose pyrophosphorylase signature 1.  GLUCOSE-1-PHOSPHATE ADENYLYLTRANSFERASE-RELATED |
| evm.model.Hap1-Super-Scaffold_100036.74 | 0.12014386492632803 | V | V | V | V | X | 484 | ENDO-1-4-BETA-GLUCANASE  Glycosyl hydrolases family 9 (GH9) active site signature 2.  ENDOGLUCANASE 11  Six-hairpin glycosidases |
| evm.model.Hap1-Super-Scaffold_100036.56 | 0.11914843783224532 | X | X | X | X | V | 182 | BCR-ASSOCIATED PROTEIN- BAP  Coil  ENDOPLASMIC RETICULUM TRANSMEMBRANE PROTEIN 3 |
| evm.model.Hap1-Super-Scaffold_100036.39 | 0.11741771868679705 | V | V | X | X | V | 191 | Vaccinia Virus protein VP39  SAM-dependent O-methyltransferase class II-type profile.  O-METHYLTRANSFERASE  CAFFEIC ACID 3-O-METHYLTRANSFERASE 1-LIKE  S-adenosyl-L-methionine-dependent methyltransferases |
| evm.model.Hap2-Super-Scaffold_100004.544 | 0.11637720642979217 | V | V | V | V | X | 99 | Activating enzymes of the ubiquitin-like proteins  NEDD8-ACTIVATING ENZYME E1 REGULATORY SUBUNIT  ThiF family  UBIQUITIN-ACTIVATING ENZYME E1 |
| evm.model.Hap1-Super-Scaffold_100036.105 | 0.11413109571296812 | V | V | X | V | V | 413 | SAWADEE PROTEIN |
| evm.model.Hap1-Super-Scaffold_100036.95 | 0.11213985175161964 | V | V | V | V | X | 523 | ADP-glucose pyrophosphorylase signature 3.  LbH_G1P_AT_C  Hexapeptide repeat proteins  GLUCOSE-1-PHOSPHATE ADENYLYLTRANSFERASE  Nucleotide-diphospho-sugar transferases  ADP-glucose pyrophosphorylase signature 2.  Spore Coat Polysaccharide Biosynthesis Protein SpsA- Chain A  glgC: glucose-1-phosphate adenylyltransferase  Trimeric LpxA-like enzymes  consensus disorder prediction  Nucleotidyl transferase  ADP-glucose pyrophosphorylase signature 1. |
| evm.model.Hap1-Super-Scaffold_100036.88 | 0.11118942366578712 | X | X | X | X | V | 408 | LYSOSOMAL ACID LIPASE-RELATED  LIPASE  Partial alpha/beta-hydrolase lipase region  Steryl_ester_lip  alpha/beta-Hydrolases  Serine aminopeptidase- S33 |
| evm.model.Hap1-Super-Scaffold_100036.29 | 0.10791899309684382 | X | X | X | X | V | 255 | Class II aaRS ABD-related  RIBOSOME BIOGENESIS PROTEIN BRX1 HOMOLOG 2  consensus disorder prediction  Brix domain profile. |
| evm.model.Hap2-Super-Scaffold_100004.491 | 0.10704299140096113 | X | X | V | V | X | 468 | RIBONUCLEASE E/G-LIKE PROTEIN- CHLOROPLASTIC  Ribonuclease E/G family  RIBONUCLEASE |
| evm.model.Hap2-Super-Scaffold_100004.510 | 0.10371731560815957 | V | V | X | X | V | 406 | Partial alpha/beta-hydrolase lipase region  LIPASE  alpha/beta-Hydrolases  LYSOSOMAL ACID LIPASE-RELATED  Steryl_ester_lip  Serine aminopeptidase- S33 |
| evm.model.Hap2-Super-Scaffold_100004.511 | 0.10339571768341126 | X | X | X | X | V | 272 | LYSOSOMAL ACID LIPASE-RELATED  alpha/beta hydrolase fold  alpha/beta-Hydrolases  LIPASE |
| evm.model.Hap2-Super-Scaffold_100004.548 | 0.10282390227875222 | V | Out CMD2 contig | X | X | V | 304 | SAM-dependent O-methyltransferase class II-type profile.  O-methyltransferase domain  Dimerisation domain  CAFFEIC ACID 3-O-METHYLTRANSFERASE 1-LIKE  Vaccinia Virus protein VP39  ""winged helix"" repressor DNA binding domain  O-mtase  O-METHYLTRANSFERASE  "Winged helix" DNA-binding domain  S-adenosyl-L-methionine-dependent methyltransferases |

**Supplemental Table 4: 81 resistance proteins of the *CMD2* region.** The resistance proteins were inferred from the CMD2 region of the 2 haplotypes of TME3. The probability of resistance was computed with prPred. Presence in 60444 genome (GCA_963409065.1 Cornet) and in previous TME3 genome (GCA_003957995.1 Kuon) (and expression (IsoSeq) were obtained after orthologous enrichment and manual analyses of phylogenetic trees. Resistance patterns of Interproscan are reported in the last column.

| **Gene**  **ID** | **prped**  **probability** | **60444**  **presence** | **NCBI**  **TME3**  **presence** | **60444**  **Isoseq** | **TME3**  **Isoseq** | **Gene**  **Length** | **Function** | **Reported**  **resistance** |
| --- | --- | --- | --- | --- | --- | --- | --- | --- |
| evm.model.Hap1-Super-Scaffold_100036.36 | 0.86 | V | X | X | V | 896 | Glumamate receptor 3.4 | Resistance to necrotrophic pathogens (Zhu et al., 2021) (74) |
| evm.model.Hap1-Super-Scaffold_100036.73 | 0.65 | V | X | X | V | 562 | NA | NA |
| evm.model.Hap2-Super-Scaffold_100004.532 | 0.53 | V | V | X | V | 613 | MORC type protein | Resistance to Turnip Crinkle Virus (TCV) in Arabidopsis, as a component of  hypersensitive response to TCV (HRT) (Koch et al., 2017) (75) |
| evm.model.Hap2-Super-Scaffold_100004.494 | 0.41 | V | V | X | V | 876 | Cytochrome P450 | Xenobiotics detoxification (Pandian et al., 2020) (76) |
| evm.model.Hap1-Super-Scaffold_100036.52.1.61231304 | 0.34 | V | V | X | V | 252 | Arogenate dehydratase | Pathway of  Phenylalanine biosynthesis, involved in sugarcane mosaic virus resistance (Yuan et al., 2019) (77) |
| evm.model.Hap1-Super-Scaffold_100036.50 | 0.18 | V | V | X | V | 403 | Hexosyltransferase | Downregulation involved in reduction to Tobacco mosaic virus resistance  (Chong et al., 2022) (78) |
| evm.model.Hap1-Super-Scaffold_100036.76 | 0.15 | V | V | X | V | 398 | NA | NA |
| evm.model.Hap2-Super-Scaffold_100004.500 | 0.12 | V | X | X | V | 523 | NA | NA |
| evm.model.Hap1-Super-Scaffold_100036.105 | 0.11 | V | V | X | V | 413 | NA | NA |

**Supplemental Table 5:  Putative resistance proteins of TME3.** The resistance proteins were inferred from the *CMD2* of the 2 haplotypes of TME3. The probability of resistance was computed with prPred. Presence (in genomes) and expression (IsoSeq) were obtained after orthologous enrichment and manual analyses of phylogenetic trees. Resistance patterns of Interproscan are available in Supplemental Table 4.

**Supplemental Table 6: Overview of CMD resistance markers used in this study.**

| Marker | Sequence | Type | Study |
| --- | --- | --- | --- |
| SSRY 28 Fw | TTGACATGAGTGATATTTTCTTGAG | SSR | Akano et al., 2002 |
| SSRY 28 Rev | GCTGCGTGCAAAACTAAAAT |  |  |
| SSRY 106 Fw | GGAAACTGCTTGCACAAAGA | SSR | Lokko et al., 2005 |
| SSRY 106 Rev | CAGCAAGACCATCACCAGTTT |  |  |
| SSR NS158 Fw | GTGCGAAATGGAAATCAATG | SSR | Okogbenin et al., 2007 |
| SSR NS158 Rev | TGAAATAGTGATACATGCAAAAGGA |  |  |
| SSR NS169 Fw | GTGCGAAATGGAAATCAATG |  |  |
| SSR NS169 Rev | GCCTTCTCAGCATATGGAGC |  |  |
| RFLP RME -1 Fw | ATGTTAATGTAATGAAAGAGC | RFLP |  |
| RFLP RME-1 Rev | AGAAGAGGGTAGGAGTTATGT |  |  |
| SSR_NS198 Fw | TGCAGCATATCAGGCATTTC | SSR | Okogbenin et al., 2012 |
| SSR_NS198 Rev | TGGAAGCATGCATCAAATGT |  |  |
| s05214_1427095_chromosomeXII_5549883_23.57_- | GCTTCAAAACACTCCAGACGCTGCACAAACGTCTCTGCTGCGAAATCCCCAGTTGAAGAATGGAGGGATTGGTTTCAACTAGGGTTTCCCTCAGGACTACTC | SNP | Rabbi et al., 2014 |
| s05214_1380239_chromosomeXII_5596739_22_- | TTCCCTTTTGCCCCATGTTAAGATGCCTCCCATTTATCTAAGGCCCAATTCTGGAATTAAGGCCAAGTTGTTTTTGTTAGAGTTGTTGTAAAAGGCTGCATT |  |  |
| s05214_1373138_chromosomeXII_5603840_23.92_- | AAAGAAAACTGCACATGCCTACCTCAACTAACTTAAAATGGTCAATATCTGGTTTACAGACGAATGCCCCAAATACCCACTGGCAAAATCTTCTCCAGACTCC |  |  |
| s05214_1273143_chromosomeXII_5703835_23.57_- | GCTGCATAACTGAGGATGATCCAGCTGGGTGTTTATTTGATAGTTGATATCACCAGCTGTATGCATGATGATTTTGGTTATTCAATCCAGTAAATTGACTCC |  |  |
| s05214_1215853_chromosomeXII_5761125_27.55_- | TTTATTAGCTCAGTTGCATCCACTCCGATTCCTTTCACCTCCATTTGATCAGTCCTTTGCAAGCGCAACAGATGCCACCCCTTAATCATCACATGTATATAC |  |  |
| s05214_1190358_chromosomeXII_5786620_27.33_- | GAAAAATAAATGATAAGAAGAAAAGGGTATGATTCAATATCCTCATCTCTTGGCTGCTTACTCTGTTTCTGCTTCTCCTCTCTTTATACAATAATGGAATAG |  |  |
| s05214_1115740_chromosomeXII_5861238_27.33_- | TTGATTTTCTTTTTTCTTTTTTCTTTTTTCTTTTTTGCCTCCATTCCTACCTGCTACGGGGTGTAATAGCTGCGCGTAGAAGTTAAATGAGAGGTAAAATTA |  |  |
| s05214_1115005_chromosomeXII_5861973_27.33_- | ATCTTCTAATGATGCCATCTGTTGCAGCAAAACCCAATTCATCAAATTCCGAATACCCACAAATATAACACATTGAAATTATATGCATACGTATACAATCCC |  |  |
| s05214_1081665_chromosomeXII_5895313_27.33_- | TAATCTCTGCCACCAGGCATAATTCACACATCTTTTCAAGAAGCCACTAAGTTCACAATGAACAAATTGCTGCATGTAAAACTTAAATGGTAACCAATGGAC |  |  |
| s05214_1081652_chromosomeXII_5895326_27.33_- | AGTTCTACAATATTAATCTCTGCCACCAGGCATAATTCACACATCTTTTCAGAGAAGCCACTAAGTCACAATGAACAAATTGCTGCATGTAAAACTTAAATG |  |  |
| s05214_1081274_chromosomeXII_5895704_27.33_- | CATACAAACCAAGGTAACGTAAATGCAGCACCTATGGAGATGGATCTGCAGATGATTGGCAACTGAAGCCTATTAGCAAGGCACCTAACCTGCTGGTCTTTA |  |  |
| s05214_1076643_chromosomeXII_5900335_27.33_- | ACCATTTTGGTCCACTTCATCCACATGGGAATGGATACGGATGAAACCAATAGTATCTGATAACTGGGCGAGCACCAGTCAAGTTCCTTTTCCCTGTTGAAT |  |  |
| s05214_1076642_chromosomeXII_5900336_27.33_- | AACCATTTTGGTCCACTTCATCCACATGGGAATGGATACGGATGAAACCAAGTGTATCTGATAACTGGGCGAGCACCAGTCAAGTTCCTTTTCCCTGTTGAA |  |  |
| s05214_1076455_chromosomeXII_5900523_27.33_- | TCTGGTATTCAACAAAGTGACAGATTGACTAGTTCCCACCTTACTAAATCAGAGTAAAAAGAAAGTAAACAAGTAGCACACCAAGTGATACTACCTCAGGCA |  |  |
| s05214_1076425_chromosomeXII_5900553_28.64_- | GCAGCCAAGGAGCCAAAATAGCTTAAATTTTCTGGTATTCAACAAAGTGACCAGAGATTGACTAGTTCCCACCTTACTAAATCAAGTAAAAAGAAAGTAAACAA |  |  |
| s05214_1041782_chromosomeXII_5935196_28.82_- | CTAATTAACATCTCTCACAGAATCCATGTGATAGTTCTTTCGTTCTTGCTTCCTTTATATGCACATATGCAGACAATCAAGTTCCAGGCAGAAACGGGAAGC |  |  |
| s05214_1011668_chromosomeXII_5965310_28.2_- | CTAGACAGGATGTGTCTTTGTTATCACCAAATAATACTGATAGTCTCTGCAGATTCCATGGTGGAGACACAAGGATATATTTGCCAAGAACATAAAGTACCG |  |  |
| s05214_981075_chromosomeXII_5995903_33.36_- | GGGCCATAGTTCCTGCGGTGAAGCCGGAGCCTAGACCATCTCCTTCCTTCGACTGCGGCAGCAGCGGCTTCTCCGGATCAAACCGCGCAGGCGGGGGCGAAG |  |  |
| s05214_980760_chromosomeXII_5996218_28.64_- | TGTTTTGGCCTTTGGCAGCGGATTATAAAACATAAATCACTGGTTTTTTCGGTTTGTTTTTTTTTTTTAAAACATAAATCACCGGTAACCATGGGCAAATGAACAAA |  |  |
| s05214_980758_chromosomeXII_5996220_26.75_- | TCTGTTTTGGCCTTTGGCAGCGGATTATAAAACATAAATCACTGGTTTTTTCGTTTTTTTTTTTTAAAACATAAATCACCGGTAACCATGGGCAAATGAACAAA |  |  |
| s05214_957480_chromosomeXII_6019498_29.27_- | ATAGGAAATAGATATTGATAGTGATGCTATGGACTGAAAGGGGGAATTGGCGTCAGCAGCATCTTAAGCGTAGTGACATAGTTGGCATATTTTTAACTAGTC |  |  |
| s05214_864519_chromosomeXII_6112459_38.83_- | TAATCTATTCTTTATTTTTTGTAATTATGGAAGTGGAAGTTTTGTAAAGAGAAGAGAGGCCGTCTTCCCATCTTCAAAATTCAAAAGAAAATAAATAAAATTAC |  |  |
| s05214_776142_chromosomeXII_6200836_38.13_- | TGTGATGTTTTTGAAGTTTTTGAGAGAGCGAGCTCGGATTATTACTACTTAGTTGTGCTCTAGCTTTGGCTGAATGCTATTTGTTTTCAGTTTCCCAGTTTT |  |  |
| s05214_755846_chromosomeXII_6221132_29.27_- | CTTGATGGTCTTTTTGGAACAGGAACTGGTTCCACTGTTTCTGTGTAAGTTATAAACTTTTTTGAGCCGTCTTTAGCAGCTTCTTTTATACAGAAATGTCGAT |  |  |
| s05214_755621_chromosomeXII_6221357_37.11_- | GTATTAAGCAGAGGGATTGGCTGGCAGGTGTGCTGGTTTAAAAGTAATTGCTTTTTGGTTGAGCATGTAGGAAATAATATTACAATCCAGTGCAAAATTTTT |  |  |
| s05214_730604_chromosomeXII_6246374_29.27_- | AAAATTTGGTATATTTGGCCCTCGTTGTATAGTTCAGGGGTAAATTGGCCTCTTTAGCCGTTACCAAACTACCTGTCTAAGAATTGCAGCCCTCCTAGTAAA |  |  |
| s05214_730219_chromosomeXII_6246759_38.13_- | TTGAGGTACTATTTTGTTCTGCCTTTCCCTATTCTCTCTCTCGGCATGCAAGATTCCATTAGGATCTGGAACTATGTTCCCATTTATGTTCTTTAGATCATAA |  |  |
| s05214_730196_chromosomeXII_6246782_38.13_- | CAGAGCTGCAAATGTCCAGTATATTGAGGTACTATTTTGTTCTGCCTTTCCGCTATTCTCTCTCTCGGCATGCAAGTTCCATTAGGATCTGGAACTATGTTC |  |  |
| s05214_719298_chromosomeXII_6257680_30.25_- | CAGAATACTCATTTTTTCCCTGTGAAGATGTGTTGCATCTTGACAGGATCCACATGATCATAGATTCATCTGCAATCTTGCAATTCTGCATTAAGCAATGCA |  |  |
| s05214_719029_chromosomeXII_6257949_30.25_- | ATTATTTGGAGCAGCAAGTGCTAGGAAACTTCTGTTTTGGAGAGCCTTAACTGGGATATCTCTCATTGTTGAGTTTTATTTATTGGTAAGTTCAACTTGGTG |  |  |
| s05214_656337_chromosomeXII_6320641_37.19_- | CCCAATGTCTGCTAACTCTAAAGGCCAAACTAAAAATTTCTCTATTTCTTCCTTCTTTTCTTCTCCAAACCTCATACATGGATGGGATAGCGACTCCATAGTG |  |  |
| s05214_656334_chromosomeXII_6320644_37.19_- | TTTCCCAATGTCTGCTAACTCTAAAGGCCAAACTAAAAATTTCTCTATTTCCTTTTCTCTTTTCTTCTCCAAACCTCATACATGGATGGGATAGCGACTCCATA |  |  |
| s05214_656308_chromosomeXII_6320670_36.65_- | ACAGACTTTAGGGTCTAAAAGCAGCGTTTCCCAATGTCTGCTAACTCTAAAGGGCCAAACTAAAAATTTCTCTATTTCTTCTCTTTTCTTCTCCAAACCTCA |  |  |
| s05214_611967_chromosomeXII_6365011_42.72_- | TTCTTCTTGTTCTCATAAGTTGAATCACCTAGTGTAGCTGAGGCGCGCCTCTGGGGGCACCTTGCGCCTGGGTGCGCCTTGGCTGCCTCGTCTCGCCTCCAG |  |  |
| s05214_472417_chromosomeXII_6504561_38.13_- | TTGTAAACATTTATAATCTGCAAGTTTAAAACGTAAATTACTTGAGCAAGACATTTCCAATTAAAAAAGAAAATCAACCATGCATGCAATGTTCTACCTTTAA |  |  |
| s05214_472404_chromosomeXII_6504574_38.13_- | TTCGTACTCCTGATTGTAAACATTTATAATCTGCAAGTTTAAAACGTAAATGTACTTGAGCAAGACTTTCCAATTAAAAAAGAAAATCAACCATGCATGCAA |  |  |
| s05214_472398_chromosomeXII_6504580_38.13_- | AGCGCTTTCGTACTCCTGATTGTAAACATTTATAATCTGCAAGTTTAAAACGGTAAATTACTTGAGCAAGACTTTCCAATTAAAAAAGAAAATCAACCATGC |  |  |
| s05214_371707_chromosomeXII_6605271_39.18_- | TACCTAGCTGAAACAGAACTTCCTGACCCCATAATATTCGCTTCTGCAGAGACCATTGTGATTGTGAAAAGTTCCTGAAAAATAATCCTGCTGTGAAAAGCC |  |  |
| s05214_371683_chromosomeXII_6605295_39.18_- | TAAGCAAGGCAGCCTATGTATACATACCTAGCTGAAACAGAACTTCCTGACTCCCATAATATTCGCTTCTGCAGAGCCATTGTGATTGTGAAAAGTTCCTGA |  |  |
| s05214_283847_chromosomeXII_6693131_43.86_- | GCAAGTCCAAAAAGCCTAATATCCAAGTAAATATTCACAACTTCATACCCGAAGAAAACTATGCTATGATACAACCTTCTTACAAGACCTGGAAAGGCCTTT |  |  |
| s05214_232019_chromosomeXII_6744959_38.13_- | TGGCTCTCTTGGACGACGGGGTAACCGAACTCTGATTGGACAATGAACAACTTACATTCCAAACTAGCTGCCATTGAAGAACTCCTTACCACTATTGCTAAA |  |  |
| s05214_232013_chromosomeXII_6744965_38.13_- | AGAATTTGGCTCTCTTGGACGACGGGGTAACCGAACTCTGATTGGACAATGTAACAACTACATTCCAAACTAGCTGCCATTGAAGAACTCCTTACCACTATT |  |  |
| s05214_232000_chromosomeXII_6744978_38.13_- | AAAATAGTGGAGAAGAATTTGGCTCTCTTGGACGACGGGGTAACCGAACTCTTGATTGGACAATGAACAACTACATTCCAAACTAGCTGCCATTGAAGAACT |  |  |
| s05214_231473_chromosomeXII_6745505_42.43_- | GATTAAAAGCCCAGCCCAGAGATTGGCCCTTAGACAATGGAAGGAATATTTCCAGAAGGTACAAGAAGAGAGGAGCGGATTGCATAGGAGCCGTTGGAACCA |  |  |
| s05214_42890_chromosomeXII_6934088_44.24_- | TATTGTGCGACAATCTACAAACGGTAAAATTATTCCTAACGTTAAATTTCTCGGCAGAACATAAATGACTAACCAGCAGCACTATAGAGTACAATGATTGCA |  |  |
| s05214_42884_chromosomeXII_6934094_42.43_- | CCATAATATTGTGCGACAATCTACAAACGGTAAAATTATTCCTAACGTTAACATTTCTGGCAGAACATAAATGACTAACCAGCAGCACTATAGAGTACAATG |  |  |
| s05214_42862_chromosomeXII_6934116_44.24_- | TTCCTCAGTGATCTCCCTTCCTCCATAATATTGTGCGACAATCTACAAACGAGTAAAATTATTCCTAACGTTAAATTTCTGGCAGAACATAAATGACTAACC |  |  |
| s05214_42565_chromosomeXII_6934413_44.24_- | AGTAGCTGAAACTGCTGCAACTTGTAGATTAGAACAGACACTTGAATCGGATGTCTGTTACTGCGACATAGTCTTTATCATCCTGTACGCCAGTTGGCTCTG |  |  |
| s05214_42557_chromosomeXII_6934421_44.24_- | TTATAAGCAGTAGCTGAAACTGCTGCAACTTGTAGATTAGAACAGACACTTAGAATCGGAGTCTGTTACTGCGACATAGTCTTTATCATCCTGTACGCCAGT |  |  |
| s05214_42485_chromosomeXII_6934493_44.24_- | TTCGGTGGACTTCTTTCACAATTTTCACATGTGTTTATGGCAGAAATTTCAGCTTTCATCTAAATTTGTATCCTTATAAGCAGTAGCTGAAACTGCTGCAAC |  |  |
| s05214_42147_chromosomeXII_6934831_44.24_- | GGATGCAGTGAAAGATCTGGATTCAGAGCAAGCTAAATCTTGTTAGAGATAGCATTGACCTTGATTTTATGAGTGTATCTTGGTCAAAGAAAGCATGATGAG |  |  |
| s05214_42144_chromosomeXII_6934834_44.24_- | AACGGATGCAGTGAAAGATCTGGATTCAGAGCAAGCTAAATCTTGTTAGAGAATACATTGACCTTGATTTTATGAGTGTATCTTGGTCAAAGAAAGCATGAT |  |  |
| s05214_42097_chromosomeXII_6934881_39.78_- | ATGTATTTTATTAGCGGCAGCAATGCATGGTACGCTCAATGTTTTACAACGAGATGCAGTGAAAGATCTGGATTCAGAGCAAGCTAAATCTTGTTAGAGATA |  |  |
| s06906_361646_chromosomeXII_7108969_45.41_- | CAAAAGAAAGAAAAACAAAGAATTATACAATTAATATTATGCCAAGGAACTATAAAAAAATGGTGATGACGGGAAAATGTACAGGATATAGTAGCAAAAGACT |  |  |
| s06906_361175_chromosomeXII_7109440_42.43_- | AATCTCTGGAGCTTCCACTTCCATACTCAGCTCCAGCCAAAATAATGGTGTCCATGTCCAGCAGTCTTGTACCTCTGAAACAGTAGAATGATCACCCAGAGT |  |  |
| s06906_314513_chromosomeXII_7156102_40.15_- | CATGATGACTTTGTCGTGTATCACAGCCAGAAAAGGAGCCAAGCATCCCCTCGTCGTGGTTCTTGATGAAGTATTTGGCAACCGGTGCTAAGCCAAAAAACC |  |  |
| s06906_282077_chromosomeXII_7188538_41.73_- | TTGTAAGAGATTTTGGAACGCTTCTTTGCCAGGGCATCACCCAATACAGATGATGCCACTTCTTCCATAATCATCATCTACAAAGACGGCAATTACCTCCCT |  |  |
| s06906_70963_chromosomeXII_7399652_52.51_- | GTCTCCGCCTCCAGCTTCTATAATCTTTTCTTATTTGTTACAGGGGTTTGGTTTTCAGATATCACTTAGCATACATAATGGGGAAGAAGCGAAAGCATAGTG |  |  |
| s06906_70962_chromosomeXII_7399653_52.51_- | CGTCTCCGCCTCCAGCTTCTATAATCTTTTCTTATTTGTTACAGGGGTTTGAGTTTCAGATATCACTTAGCATACATAATGGGGAAGAAGCGAAAGCATAGT |  |  |
| s06906_70919_chromosomeXII_7399696_51.59_- | TCGACGCAGCAAACAGGTCTGCTTCCTCCTCCTCTTTCGCCGCCGTCTCCGCCCTCCAGCTTCTATAATCTTTTCTTATTTGTTACAGGGGTTTGGTTTCAG |  |  |
| s06906_39739_chromosomeXII_7430876_52.51_- | TTTGGCACTTCAATTGTAGAGGTGAATACTCTGTCAAATCGGGCTATAGAAGTGGCCAGACAAATCAAGCTGCAACAAATGCCTAGTTCTAGTACTTCGGGC |  |  |
| s06906_39732_chromosomeXII_7430883_52.51_- | ACTTGGCTTTGGCACTTCAATTGTAGAGGTGAATACTCTGTCAAATCGGGCATATAGAATGGCCAGACAAATCAAGCTGCAACAAATGCCTAGTTCTAGTAC |  |  |
| s06906_39485_chromosomeXII_7431130_47.64_- | GAGAGTGGATTCAAAATGTTTCGGTTGAAAAGAAAAGCTTCTCTCAAAAGGCAAATAAAGAGATTTTAATCAAAGCATTTGCTTCGGCCATCTCAATCTACA |  |  |
| s06906_39459_chromosomeXII_7431156_52.51_- | AGCAGCAAGTTTTTAGCTTCCTCAAAGAGAGTGGATTCAAAATGTTTCGGTATGAAAAGAAAAGCTTCTCTCAAAAGGAAATAAAGAGATTTTAATCAAAGC |  |  |
| S12_7828503_0.69_Rabbi_et_al_2020 | CCTCCTTATAGTTTAAAAGTGTAGAATTTAGTGTATTGGGATCTTTCAAGTAATGGGTTGTTTACTATTAAGTTTGCATATGCTGCATTAGCTAATAGCTTGTGGGGTTTGGAAAATAGTGAATGGAAGTTTGCTTGGAGTTGAGCTGGCACTAAGAGTATTTATACCTTTTTCTAATTAGTGTAGTACGAGAAGTTGATA | SNP | Rabbi et al., 2020 |
| S12_7828514_0.27_Rabbi_et_al_2020 | TTTAAAAGTGTAGAATTTAGTGTATTGGGATCTTTCAAGTAATGGGTTGTTTACTATTAAGTTTGCATATGCTGCATTAGCTAATAGCTTGTGGGGTTTGGAAAATAGTGAATGGAAGTTTGCTTGGAGTTGAGCTGGCACTAAGAGTATTTATACCTTTTTCTAATTAGTGTAGTACGAGAAGTTGATAGCAGCGGATGG |  |  |
| S12_7842538_5.96_Rabbi_et_al_2020 | CGAGTAGCGGATTGCTCAACCTCTTCAAGTATAATTCTATTAAAAATTAAAGAAGAAACAAAAATTGGCAGCAACCCTTGTGCCTGAATGGTGGTGTAAAATTGAATATTTATAAATTTCTATTCACAGTTAAGAGAAAAACTAAACTTTGGAAATGGACATATATATAAATTTTAGTAACACACACATAACAAAAAAAGA |  |  |
| S12_7842568_17.24_Rabbi_et_al_2020 | ATAATTCTATTAAAAATTAAAGAAGAAACAAAAATTGGCAGCAACCCTTGTGCCTGAATGGTGGTGTAAAATTGAATATTTATAAATTTCTATTCACAGTTAAGAGAAAAACTAAACTTTGGAAATGGACATATATATAAATTTTAGTAACACACACATAACAAAAAAAGAACACGGACAAACCTTCCTTCATCCTCCATA |  |  |
| S12_7842654_6.57_Rabbi_et_al_2020 | TTTCTATTCACAGTTAAGAGAAAAACTAAACTTTGGAAATGGACATATATATAAATTTTAGTAACACACACATAACAAAAAAAGAACACGGACAAACCTTCCTTCATCCTCCATATCTTTTTAACAATGCTTGAAACTGCAAGTTTTGCAGCATCTTGCATGGCAAGACCCTTCATTGACAATCCTATATATTGGAAATGG |  |  |
| S12_7842657_0.93_Rabbi_et_al_2020 | CTATTCACAGTTAAGAGAAAAACTAAACTTTGGAAATGGACATATATATAAATTTTAGTAACACACACATAACAAAAAAAGAACACGGACAAACCTTCCTTCATCCTCCATATCTTTTTAACAATGCTTGAAACTGCAAGTTTTGCAGCATCTTGCATGGCAAGACCCTTCATTGACAATCCTATATATTGGAAATGGCAA |  |  |
| S12_7842670_16.26_Rabbi_et_al_2020 | AGAGAAAAACTAAACTTTGGAAATGGACATATATATAAATTTTAGTAACACACACATAACAAAAAAAGAACACGGACAAACCTTCCTTCATCCTCCATATCTTTTTAACAATGCTTGAAACTGCAAGTTTTGCAGCATCTTGCATGGCAAGACCCTTCATTGACAATCCTATATATTGGAAATGGCAAGTGTGCTCTTTAT |  |  |
| S12_7849710_0.07_Rabbi_et_al_2020 | TTCAATGAATAAAAATTCATCTATGAATAGAGCAGCAGCGTCTGATCAGGAGAGCAGTGGTTGGACAGCTTATTTTGAAGATTTCTCTACCCATAGAGATCAAGATGATTGTTTCTCTTCTGGTTTTGGTAGCTCTTCAATGGTGTCTGATGCCGCATCTTATCCTGCATGGAAATCGTCAACTCATCATAATTATAACCA |  |  |
| S12_7874196_19.69_Rabbi_et_al_2020 | GGTCGGATTTCCGGGTCGTTACAATGAGTTTGGGAGACAGTAGCTGCTATGGCTTTGCCCTTGTTTCTATTCTTGAGTTCCATAAACCATTCAGGATATCTATGCACTCTAAAGCATGTATCCTTAGTATGACCAGTTGATTTGCAATGAATGCAAATCCTATCATCTTTCTTCCCAGATACTCCCTTCTTGAAGGCAATC |  |  |
| S12_7874273_15.64_Rabbi_et_al_2020 | TTCCATAAACCATTCAGGATATCTATGCACTCTAAAGCATGTATCCTTAGTATGACCAGTTGATTTGCAATGAATGCAAATCCTATCATCTTTCTTCCCAGATACTCCCTTCTTGAAGGCAATCTTGTTATAACCTATGTTACTCTTAGCAGCAAAAATATTGTTAATATCATCACCAGGAAGATTATGCACTTCTCTTTG |  |  |
| S12_7874328_4.68_Rabbi_et_al_2020 | CCAGTTGATTTGCAATGAATGCAAATCCTATCATCTTTCTTCCCAGATACTCCCTTCTTGAAGGCAATCTTGTTATAACCTATGTTACTCTTAGCAGCAAAAATATTGTTAATATCATCACCAGGAAGATTATGCACTTCTCTTTGTTTCTTAATCCTTAAAATCATTGAATATGCTTTGTTAACACTGGGCAAGGGATCT |  |  |
| S12_7874331_4.64_Rabbi_et_al_2020 | GTTGATTTGCAATGAATGCAAATCCTATCATCTTTCTTCCCAGATACTCCCTTCTTGAAGGCAATCTTGTTATAACCTATGTTACTCTTAGCAGCAAAAATATTGTTAATATCATCACCAGGAAGATTATGCACTTCTCTTTGTTTCTTAATCCTTAAAATCATTGAATATGCTTTGTTAACACTGGGCAAGGGATCTAGC |  |  |
| S12_7874349_4.55_Rabbi_et_al_2020 | CAAATCCTATCATCTTTCTTCCCAGATACTCCCTTCTTGAAGGCAATCTTGTTATAACCTATGTTACTCTTAGCAGCAAAAATATTGTTAATATCATCACCAGGAAGATTATGCACTTCTCTTTGTTTCTTAATCCTTAAAATCATTGAATATGCTTTGTTAACACTGGGCAAGGGATCTAGCAGCAAGATTTGATTCTTT |  |  |
| S12_7874475_17.03_Rabbi_et_al_2020 | TTCTTAATCCTTAAAATCATTGAATATGCTTTGTTAACACTGGGCAAGGGATCTAGCAGCAAGATTTGATTCTTTGCATTATCTTAGCTATCATTTAAATCCATTATAAATTGTATGAGCCTATCATCATTCTCCATTTCACACAAATCCTTTGCAGCACCACACTCACACATAGGCAGAGGCCCCAAGCATGGAAGTTCA |  |  |
| S12_7926088_36.43_Rabbi_et_al_2020 | TGCTAAAAGAAAAGGTCAAAAACTTTCAGCCAAGTTTTATGGACCTTTCGTGGTTCTTGAGCAAATCGGTTCCATGGCTTATAAGCTAGACTTACCTGCACACTCAAAGCTGCATCCTATTTTCCATGTTTCCACCCTCAAATGGTATCACAAAGGACAAGATTCTTGTACTCCAATGCTGCCACCAACTCCACCTGATGT |  |  |
| S12_7926132_111.76_Rabbi_et_al_2020 | CTTTCGTGGTTCTTGAGCAAATCGGTTCCATGGCTTATAAGCTAGACTTACCTGCACACTCAAAGCTGCATCCTATTTTCCATGTTTCCACCCTCAAATGGTATCACAAAGGACAAGATTCTTGTACTCCAATGCTGCCACCAACTCCACCTGATGTTCCTCTTCAACCTCTGGCTGTTTTAGACCAGCGTATTATGGCCA |  |  |
| S12_7926163_110.47_Rabbi_et_al_2020 | GGCTTATAAGCTAGACTTACCTGCACACTCAAAGCTGCATCCTATTTTCCATGTTTCCACCCTCAAATGGTATCACAAAGGACAAGATTCTTGTACTCCAATGCTGCCACCAACTCCACCTGATGTTCCTCTTCAACCTCTGGCTGTTTTAGACCAGCGTATTATGGCCAGACAACTTGAGATTTTAGTCCATTGGTGTGC |  |  |
| S12_7954248_100.35_Rabbi_et_al_2020 | CCATGGATTCACTTTTGCTCAAGATTTTGCTGATTCAATGGTTAAACTTGGTGATGTTGGCGTCCTCACTGGCTGCCAAGGTGAAATCAGGAAACGCTGCACTTTCGTCAATTAATTATTCCCCATATTTCTTCTTCTGCTTCTCCTAATAAAATAATGATTTGATCAATCCATTGATCGTATTGCAGCAAAGTTACATGT |  |  |
| S12_7954303_103.86_Rabbi_et_al_2020 | GTTGGCGTCCTCACTGGCTGCCAAGGTGAAATCAGGAAACGCTGCACTTTCGTCAATTAATTATTCCCCATATTTCTTCTTCTGCTTCTCCTAATAAAATAATGATTTGATCAATCCATTGATCGTATTGCAGCAAAGTTACATGTTTGTGTAAATTTCTTTTCAAATTTTTGTATTCTTTTTTTTTTTTTTTTAGGTTTT |  |  |
| S12_7961675_29.87_Rabbi_et_al_2020 | CTAGACCTTCACCTTCATGGTTTTGATGCAAAATGTCACATACCTCTGTCCACTATGTTGCTGCTAATAAGTAAAGACGATCTGAGTCGAAGTGGCGAGTTTATCCATATAGACACTGCATGGGGGTAGGAGAAAAAGGCATGATCAATTGTTTTCTTTTCACCACAGAATGCATAATGTGGATTCAAAACTTGAATTTGA |  |  |
| S12_7961873_11.06_Rabbi_et_al_2020 | TGACGATATAAAATCTCCTTCACAGGAAGCTGTTCCACATAAAGAAGCTAAAGAAACATCCGAAGCTTGGTAGAAAGGTTAAGTTTCCAGAATTGTTTCTAAAAATGGGAAGAAAGGTTATATGGAAGCTGGCCCGAAGTACTAGAACTAGGCATTTGTTGCAGCTTGATTTGTCTGGCCATTCTATAGCCCGATTTGACA |  |  |
| S12_7961874_11.1_Rabbi_et_al_2020 | GACGATATAAAATCTCCTTCACAGGAAGCTGTTCCACATAAAGAAGCTAAAGAAACATCCGAAGCTTGGTAGAAAGGTTAAGTTTCCAGAATTGTTTCTAAAAATGGGAAGAAAGGTTATATGGAAGCTGGCCCGAAGTACTAGAACTAGGCATTTGTTGCAGCTTGATTTGTCTGGCCATTCTATAGCCCGATTTGACAG |  |  |
| S12_7961875_11.37_Rabbi_et_al_2020 | ACGATATAAAATCTCCTTCACAGGAAGCTGTTCCACATAAAGAAGCTAAAGAAACATCCGAAGCTTGGTAGAAAGGTTAAGTTTCCAGAATTGTTTCTAAAAATGGGAAGAAAGGTTATATGGAAGCTGGCCCGAAGTACTAGAACTAGGCATTTGTTGCAGCTTGATTTGTCTGGCCATTCTATAGCCCGATTTGACAGA |  |  |
| S12_7961876_11.43_Rabbi_et_al_2020 | CGATATAAAATCTCCTTCACAGGAAGCTGTTCCACATAAAGAAGCTAAAGAAACATCCGAAGCTTGGTAGAAAGGTTAAGTTTCCAGAATTGTTTCTAAAAATGGGAAGAAAGGTTATATGGAAGCTGGCCCGAAGTACTAGAACTAGGCATTTGTTGCAGCTTGATTTGTCTGGCCATTCTATAGCCCGATTTGACAGAG |  |  |
| S12_7961878_10.97_Rabbi_et_al_2020 | ATATAAAATCTCCTTCACAGGAAGCTGTTCCACATAAAGAAGCTAAAGAAACATCCGAAGCTTGGTAGAAAGGTTAAGTTTCCAGAATTGTTTCTAAAAATGGGAAGAAAGGTTATATGGAAGCTGGCCCGAAGTACTAGAACTAGGCATTTGTTGCAGCTTGATTTGTCTGGCCATTCTATAGCCCGATTTGACAGAGTA |  |  |
| S12_7962234_32.95_Rabbi_et_al_2020 | CTGGTAAATGGAAGCAAGACATGGTGTAGATTGAGATGGCCGAAGCAAATGCTTTGATTAAAATCTCTTTATTTCCTTTTGAGAGAAGCTTTTCTTTTCAACCGAAACATTTTGAATCCACTCTCTTTGAGGAAGCTAAAAACTTGCTGCTTAGAACGAGGGACACTAGAGGGTAAACCTAAGAATTTGTCTTGAGTGTTA |  |  |
| S12_8038259_7.34_Rabbi_et_al_2020 | AGGAACTGATGGCAAGACTACCCTCTTGCACTTTGTCATCCAGGAGATTATCCGTTCTGAAGGTAAACGAGCTCTCCGCAGCATAAAAGCGAGCCAGAGTACTTGTAGTTTAAAGTCAGAGGATTTGGTTGAGGATACTAATCAGTCATCAGAACACTATCGTAACCTGGGTCTTAAGGTTATTTCAGGCTTAAGCAATGA |  |  |
| S12_8038319_28.2_Rabbi_et_al_2020 | AGGTAAACGAGCTCTCCGCAGCATAAAAGCGAGCCAGAGTACTTGTAGTTTAAAGTCAGAGGATTTGGTTGAGGATACTAATCAGTCATCAGAACACTATCGTAACCTGGGTCTTAAGGTTATTTCAGGCTTAAGCAATGAATTAGAAGACGTAAAAAATGCAGCAGCAGTAGATGCTGACGTCCTAACATCTACAGTTTC |  |  |
| S12_8039677_22.6_Rabbi_et_al_2020 | TTTCAGGTGGCGTGGATATCCTGGAGGCTTCATAACTCTATTGTACACTTTACACTGAGAAAAAAGATTCGCATACCTCAATCCTGCCTGTGGCAGCATACGTGCATGTCATTCTTGAAATTCCTTGATTTAAATGATGCTTAAAATTTTAGGGAGGTAATTTCATTATAGAAAGAGATTCACTTGGAGGAGTTCTTTGTT |  |  |
| S12_8039686_20.83_Rabbi_et_al_2020 | GCGTGGATATCCTGGAGGCTTCATAACTCTATTGTACACTTTACACTGAGAAAAAAGATTCGCATACCTCAATCCTGCCTGTGGCAGCATACGTGCATGTCATTCTTGAAATTCCTTGATTTAAATGATGCTTAAAATTTTAGGGAGGTAATTTCATTATAGAAAGAGATTCACTTGGAGGAGTTCTTTGTTAATTCCTAC |  |  |
| S12_8071420_30.62_Rabbi_et_al_2020 | AATCCTCTACTCCTCTCCTATATAATCTCTTTCCACCTACTGCCGAATTAAAACCCTCCATGGCTATTAAGCGTCTATCTGAAAATCCGCCACCTGCTGCTTCCTCTTCGGAAGAAGAGGAGGAGGAAGAAAACGACTCTGTGGAGAAAAACGACAGTGAGGATGAGCAAAAGGACGTCGGTGATGGAGACGACGATGAGG |  |  |
